# Developing process guidelines for trauma care in the Netherlands for severely injured patients: results from a Delphi study

**DOI:** 10.1186/1472-6963-13-79

**Published:** 2013-03-03

**Authors:** Elisabeth Maria Hoogervorst, Eduard Ferdinand van Beeck, Johan Carel Goslings, Pieter Dirk Bezemer, Joost Jan Laurens Marie Bierens

**Affiliations:** 1 Department of Anaesthesiology, VU University Medical Center, Amsterdam, the Netherlands; 2 Current address: Department of Public Health, Erasmus MC, University Medical Center, Rotterdam, the Netherlands; 3 Department of Public Health, Erasmus MC, University Medical Center, Rotterdam, the Netherlands; 4 Department of Surgery, Academic Medical Center, Amsterdam, the Netherlands; 5 Department of Epidemiology and Biostatistics, VU University Medical Center, Amsterdam, the Netherlands; 6 Simon van Ooststroomhof 1, Oegstgeest, 2341 KE, the Netherlands

## Abstract

**Background:**

In organised trauma systems the process of care is the key to quality. Nevertheless, the optimal process of trauma care remains unclear due to lack of or inconclusive evidence. Because monitoring and improving the performance of a trauma system is complex, this study aimed to develop consensus-based process guidelines for trauma care in the Netherlands for severely injured patients.

**Methods:**

A five-round Delphi study was conducted with 141 participants that represent all professions involved in trauma care. Sensitivity analyses were carried out to evaluate whether consensus extended across all professions and to detect possible bias.

**Results:**

Consensus was reached on 21 guidelines within 4 categories: timeliness, actions, competent teams and interdisciplinary process. Timeliness guidelines set specific critical limits and definitions for 10 time intervals in the time period from an emergency call until the patient leaves the trauma room. Action guidelines reflect aspects of appropriate care and strongly rely on the international Advanced Trauma Life Support principles. Competence guidelines include flow charts to assess the competence of prehospital and emergency department teams. Essential to competent teams are education and experience of all team members. The interdisciplinary process guideline focuses on cooperation, communication and feedback within and between all professions involved. Consensus was extended across all professions and no bias was detected.

**Conclusions:**

In this Delphi study, a large expert panel agreed on a set of guidelines describing the optimal process of care for severely injured trauma patients in the Netherlands. In addition to time intervals and appropriate actions, these guidelines emphasise the importance of team competence and interdisciplinary processes in trauma care. The guidelines can be seen as a description of a best practice and a new field standard in the Netherlands. The next step is to implement the guidelines and monitor the performance of the Dutch trauma system based on the guidelines.

## Background

Quality of trauma care can be defined as achieving the best possible outcome for a given set of clinical circumstances [1]. Quality can be assessed by evaluating the structure of the trauma system, the process of trauma care and the effect of trauma care on the health status of the patient, or the outcome [2]. Worldwide, outcome is well documented in various trauma registries. The structure needed to provide trauma care with the lowest mortality rates is secured in regional trauma systems that have been implemented in many countries. Positive effects of this organisational structure on survival rates have been clearly demonstrated [3-7]. However, as the most optimal structure can only affect outcome through the processes of care that it enables [1], the next step in quality improvement is to describe the processes that would lead to the best possible outcome when applied within the defined structure. The optimal processes should not only influence mortality, but should also positively affect functional outcome and health-related quality of life [8,9].

Although the processes of trauma care are important, only a few process guidelines in this field are available [10,11] and it is largely unknown whether these are positively related with either survival or functional outcome [12-17]. Monitoring and improving the performance of a trauma system in this situation is an arduous task. In 2009 the Cochrane collaboration attempted to determine the effectiveness of using audit filters for improving processes of care and clinical outcomes in trauma systems; however, they found no studies to include [18]. Therefore, as scientific evidence is either absent or inconclusive, process guidelines have to be developed based on expert opinion.

When guidelines are adopted and supported by all professions they can be interpreted as the description of a best practice and as a new field standard. Eventually, guidelines need to be implemented and employed in order to monitor the performance of a trauma system.

We conducted a Delphi study to develop process guidelines for quality of trauma care in the Netherlands for severely injured trauma patients. The primary aim was to compose process guidelines with an expected positive relation with survival and/or functional outcome which are supported by an expert panel representing all professions that collaborate in regional trauma care. The second aim was to determine whether panel members from different settings and/or occupations would support the same guidelines.

## Methods

### Study design

The Delphi procedure uses a series of structured questionnaires to transform opinion into group consensus. Results of every questionnaire are shared with panelists and converted to a subsequent questionnaire or round [19-21]. Our Delphi procedure consisted of 5 structured questionnaires sent to the complete panel in September 2005, December 2005, April 2006, October 2006 and April 2007. The study was combined with a Nominal Group Technique (NGT) meeting in which the participants met to discuss the process and resolve uncertainty or any ambiguities in the questionnaires.

To avoid imposing a specific view and preconceptions of process guidelines for trauma care [21], every questionnaire was primarily composed by a methodologist with knowledge of current literature, protocols and legislation but without any clinical experience in trauma care (EMH).The draft questionnaires were reviewed on relevance and methodological quality by a steering committee, composed of clinical professors in traumatology and emergency medicine, and university healthcare researchers. The systematic procedure resulted in consistently structured and unambiguous questionnaires covering all items of interest.

Each questionnaire was analysed separately and processed into a feedback report and a subsequent questionnaire, both of which were sent by email to the entire Delphi panel. In addition to the results from previous questionnaires, additional input for questionnaires was gathered from scientific literature.

Experts were explicitly asked to answer questionnaires with the care processes in mind that would elicit the best possible survival chances and functional outcome for the severely injured trauma patient.

As our study focused on reaching consensus in an expert panel, the institutional review board of the VU University Medical Center (METc VUmc) decided that study approval was not necessary.

### Setting

The study was carried out in the Netherlands, a country with 16.4 million inhabitants and a population density of 486 persons per square kilometer. The Dutch Emergency Medical System (EMS) is organised into 11 trauma regions, according to international principles with regard to the optimal structure.

Each region has at least one level 1 trauma centre, several level 2 and 3 hospitals for trauma care, one or more ambulance dispatch centres, and one or more public or private ambulance services. Each trauma region supports a Mobile Medical Team (MMT) consisting of a physician and a nurse experienced in delivering complex care at the scene of the accident to a severely injured patient. The MMT is primarily dispatched in conjunction with an ambulance when the patient meets criteria with a high possibility of severe injury. In addition, secondary dispatch of a MMT can be requested if an ambulance arrives alone at the scene and severe injuries are observed by the ambulance crew.

All trauma care providers are trained according to Prehospital Trauma Life Support (PHTLS) and Advanced Trauma Life Support (ATLS) principles. During this study there were no interventions or major changes in training, procedures or legislation related to the Dutch EMS system.

### Selection of participants

Selection aimed to include executives, physicians and nurses working at dispatch centres, prehospital care services and emergency departments in sufficient numbers to extract professional opinions of all subgroups [21]. A total of 142 opinion leaders in trauma care representing all trauma regions in the Netherlands were asked to participate in the study; of these, 141 were willing to participate. The panel included 37 executives, 39 physicians and 65 nurses. Of the panelists, 26 were employed at a dispatch centre, 60 at a prehospital care service and 45 at an emergency department. The panel members had, on average, a working experience of 13.2 (SD 7.9) years in trauma care and a mean age of 43.8 (SD 7.3) years (Table 1).

**Table 1:** Panel members and average number of working years (experience) in trauma care, by occupation and setting

		***Occupation***						***Total***	
		***Executive***		***Physician***		***Nurse***			
		**No.**	**Experience (years)**	**No.**	**Experience (years)**	**No.**	**Experience (years)**	**No.**	**Experience (years)**
***Setting***	Dispatch centre	9	22.1	4	9.3	13	7.4	26	12.6
	Prehospital care	10	15.5	11	13.8	29	14.0	60	14.2
	Emergency department	18	14.7	24	11.3	23	12.9	45	12.9
*Total*		37	16.5	39	11.8	65	12.4	141	13.3

During the Delphi procedure, 14 panel members indicated that they were no longer able to participate. Reasons for withdrawal were personal circumstances (n=2) or lack of time (n=12).

### Methods of measurement

**Figure 1:**
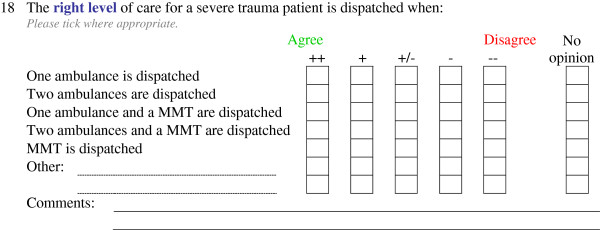
Example of a matrix question from questionnaire 3 containing several Likert items.

The opinions of experts were measured using a 5-point Likert scale ranging from ‘strongly agree’ to ‘strongly disagree’ with a separate ‘no opinion’ option. The ‘no opinion’ option was included because it was expected that some experts would encounter questions beyond the scope of their own specific field of expertise. It was hypothesised that the ‘no opinion’ option would be used most frequently by panel members employed at dispatch centres for questions about the emergency department, and vice versa. Items that related to the same question were arranged in one matrix. For each matrix, panelists could add a comment about their answers, or could add extra items. Figure 1 shows a representative example of a matrix question.

### Data collection and processing

All questionnaires were sent by email (in Word format) and most were returned by email. Questionnaires returned by post were manually converted to a Word document, double checked and processed in the same was as the digitally returned questionnaires by the first author (EMH). All answers, clarifications, added indicators and arguments were directly copied in an Excel database (Microsoft Excel version 2003, Redmond, WA, USA). Frequencies of answers and percentages of agreement and disagreement were calculated with Excel. The excel database was imported in SPSS (version 17.0, Chicago, IL, USA) for the sensitivity analysis.

### Analysis

In total, 706 items were measured on the Likert scale. Consensus was reached on an item when 70% or more of the respondents answered the item with ‘strongly agree’ or ‘agree’ (respondents not answering or with no opinion were omitted). Items passing the 70% limit of agreement were included in the feedback report to the Delphi panel and were further examined in the subsequent round. The 65-70% threshold used in our consensus definition was based on literature on the Delphi Method [21]. Qualitative results included all the free texts written by respondents in response to the questionnaire. Text was sorted by subject by one researcher (EMH) and checked by a second researcher (JJLMB); differences in opinions were solved through discussion between the two researchers. Ideas suggested by two or more respondents were included in the subsequent round. Sensitivity analysis was performed to explore consensus across the groups of panel members by analysing average item scores. In addition, possible bias caused by the use of the ‘no opinion’ option, missing values and selective withdrawal was analysed. The 706 items were independently classified by two researchers into 6 distinct domains; one of these researchers was not actively involved in the study at any time. The domains were General (n=43), Dispatch (n=33), Dispatch and Prehospital care (n=105), Prehospital care (n=222), Prehospital care and Emergency Department (n=67) and Emergency Department (n=236). Initial agreement between the researchers was good (kappa 0.75). Differences in domains were discussed and solved resulting in unanimous agreement. For every domain of items, the average Likert score was calculated. We also calculated the percentage of Likert items answered with ‘no opinion’ the percentage of Likert items that were left empty, and the percentage of Likert items with no response (due to not returned questionnaires). There were no missing values. In order to analyse the potential effect of selection bias caused by the reduced response rates in the final round, a t-test was performed to compare average domain scores of the respondents that also filled out questionnaire 5 and the respondents that filled out questionnaires 1 to 4 only. Analysis of variance (ANOVA) was performed for two separate factors (independent variables) describing the Delphi panelists by occupation (levels: executive, physician, nurse) and setting (levels: dispatch, prehospital care, emergency department). These analyses were performed with the average item score in every domain, and the percentage of ‘no opinion, ‘empty’ or ‘no response’ in every domain. Bonferonni was chosen as post-hoc test and the significance level was set at 0.05.

## Results

In round one (response rate 87%; 122/141), round two (response rate 75%; 103/137) and round three (response rate 70%; 93/132) the main aim was to develop the specific items of each guideline for which consensus was to be achieved. Round four (response rate 57%; 74/130) provided further details on these guidelines, specifically regarding acceptable limits for each guideline. The NGT meeting (n=12) focused on discussing 21 items that needed clarification. Round five (response rate 48%; 61/127) aimed to acquire the panel’s approval of the NGT results and achieve final consensus.

In round 1, four categories of guidelines were selected: time intervals, actions, competent teams and interdisciplinary process. The results of the subsequent Delphi rounds were organised according to these four categories.

### Time intervals

The critical limits for ten time intervals were developed in the final two rounds of the Delphi study. Consensus was reached on a limit when 70% or more of the respondents answered the question with ‘strongly agree’ or ‘agree’ on a 5-point Likert scale. Table 2 shows the consensus reached on the time intervals, i.e. the starting point, endpoint, critical limits or maximal duration in minutes, and their definitions. Critical limits for the time spent by the prehospital team on the accident location were only considered relevant when a ‘scoop and run’ strategy was performed. This strategy includes a rapid transportation of the patient to a hospital without attempting a major intervention at the scene; this is usually advocated for patients with severe haemorrhage or traumatic brain injury [22-24].

**Table 2:** Starting point, endpoint, maximal duration in minutes and definition of 10 time intervals selected as guidelines for severe trauma patients

**Time**			
Time intervals for dispatch setting			
	Start	End	Minutes
1	112 call at dispatch	Departure of ambulance	2
2	112 call at dispatch	Departure of MMT	2
3	Request for Mobile Medical Team (MMT)	Departure of MMT	2
Time intervals for dispatch and prehospital setting			
	Start	End	Minutes
4	112 call at dispatch	Arrival of ambulance on scene	10
5	112 call at dispatch	Arrival of MMT on scene	15
6	Request for MMT	Arrival of MMT on scene	15
*7*	*112 call at dispatch*	*Arrival at the emergency department*	*30*
Time intervals for prehospital setting			
	Start	End	Minutes
*8*	*Arrival of ambulance on scene*	*Departure of patient*	*10*
*9*	*Arrival of MMT on scene*	*Departure of patient*	*10*
Time intervals for emergency department (ED) setting			
	Start	End	Minutes
10	Arrival at the trauma room	Departure from trauma room	30
**Definitions of time points**			
Point in time		The moment that the:	
112 call at dispatch		dispatch nurse picks up the phone.	
Request for MMT		dispatch nurse picks up the phone to receive the request	
Departure of ambulance		ambulance departs to the accident location	
		Departure of MMT		MMT departs to the accident location	
Arrival of ambulance on scene		ambulance nurse starts delivering care to the patient		
Arrival of MMT on scene		MMT members start delivering care to the patient		
Departure of the patient		patient leaves the accident location		
Arrival of the patient at the ED		ED team starts delivering care to the patient		
Departure from the trauma room		patient definitively leaves the trauma room		

Guideline 7, 8 and 9 are only considered relevant when a ‘scoop and run’ strategy is performed at the accident location.

It was agreed that the ambulance and MMT should leave for the accident location within 2 min after the dispatch nurse answered the phone. In less than 10 min the ambulance should arrive at the accident location and the crew should start delivering care to the patient. Less than 5 min later the MMT should be at the scene. When a ‘scoop and run policy’ is performed the patient should be transported from the accident location within 10 min after arrival of the ambulance. The total duration from national emergency call until arrival of the patient at the trauma room of the emergency department (ED) should not exceed 30 min. The patient should spend maximally 30 min in the trauma room. The total time spent from national emergency call until departure from the ED should not exceed 60 min. (In trauma systems where a physician is always dispatched to the accident location guidelines 1, 4 and 8 are not relevant).

### Actions

Consensus was reached on 8 actions as process guidelines for quality of trauma care. Table 3 provides an overview of the action guidelines and definitions. It was agreed that the dispatch centre should send both an ambulance and an MMT to every severe trauma patient. On scene, the prehospital team should undertake all actions to get the patient stabilised according to the ABCDE approach (stands for Airway, Breathing, Circulation, Disability, Exposure and Environment). The acronym represents a quick and efficient way to assess and stabilise vital functions in trauma patients and is taught worldwide in ATLS courses. ABCDE stabilisation in the prehospital setting includes two additional actions: application of a cervical collar and immobilisation on a backboard. The prehospital team should compile a provisional/working diagnosis and transport the patient directly to a level 1 trauma centre.

**Table 3:** Description and definition of 8 appropriate actions selected as process guidelines for severe trauma patients

**Actions**	
Actions for dispatch setting	
11	One ambulance and a Mobile Medical Team (MMT) are dispatched
Actions for prehospital setting	
12	The patient is ABCDE stabilised before the hospital is reached
13	A provisional/working diagnosis is formulated before the hospital is reached
14	The patient is transported to a level 1 trauma centre
Actions for emergency department (ED) setting	
15	A complete trauma team is present in the trauma room when the patient arrives
16	The patient is ABCDE stabilised in the trauma room
17	A complete trauma series X-rays is made in the trauma room
18	A provisional diagnosis is confirmed or reformulated in the trauma room
**Definitions**	
	Includes:
ABCDE stabilisation in the prehospital setting	Manual clearance of the airway, application of a cervical collar, application of oxygen, staunching the flow of blood, insertion of two intravenous lines, administering intravenous fluids, immobilisation on a backboard, connection of the patient to a monitor and covering of the patient.
ABCDE stabilisation in the trauma room setting	Manual clearance of the airway, intubation, application of oxygen, staunching the flow of blood, insertion of two intravenous lines, administering intravenous fluids, pain medication, connection of the patient to a monitor and covering of the patient
Prehospital working diagnosis	ABCDE (Airway and cervical spine, Breathing, Circulation, Disability, Exposure and Environment), symptoms, anatomical location of injury, trauma mechanism, Glasgow Coma Scale (GCS) score, Revised Trauma Score (RTS)
Trauma room working diagnosis	Prehospital working diagnosis extended with:
	1.Findings on the trauma series X-rays
	2.Pediatric Trauma Score for juvenile patients
Complete trauma team	2 ED nurses, a trauma surgeon, an anesthesiologist, a radiologist and an X-ray laboratory assistant
Complete trauma series Rontgen	Cervical spine, chest and pelvis

At the trauma room of the ED the complete trauma team should be ready to undertake all necessary actions to keep or get the patient ABCDE stabilised. When necessary actions were not performed in the prehospital setting, they should be performed at the ED. Actions that should only be performed in the trauma room are intubation and application of pain medication. The patient should undergo a complete trauma series of X-rays; these results are evaluated by a physician and used in confirming or reformulating the working diagnosis.

### Competent teams

Consensus was reached on two competent team guidelines that indicate that all professionals involved in trauma care should have completed specific trauma courses in addition to their professional education. They should be experienced in delivering care to a severe trauma patient and should have worked in trauma care for at least 1.5 years.

**Figure 2:**
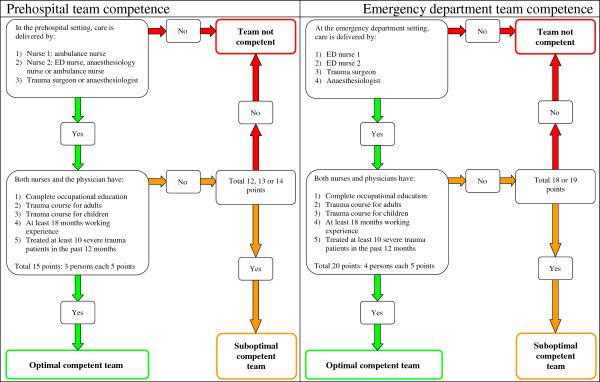
Flowcharts to determine whether the prehospital and emergency department teams are competent.

During the NGT meeting flow charts were composed to determine whether the prehospital team and the ED teams were considered competent (Figure 2). The flow charts were approved by the entire panel in round 5. Criteria for the assessment of team competence are the composition of the team, and the level of training and experience of all team members. At the accident location the patient should be taken care of by two nurses and a physician; together they are considered an optimal competent team if they all fulfill the criteria for training and experience (Figure 2, left side). At the ED two nurses and two physicians should deliver the necessary care to the patient; together they should form a competent team (Figure 2, right side).

### Interdisciplinary process

Consensus was reached on one interdisciplinary process guideline. It was agreed that, during or shortly after treating the severe trauma patient, at least 80% of all 7 team members (as named in the competent team flowcharts) should judge cooperation, communication and feedback within and between teams as satisfactory. This implies that the guideline is met when at most 1 of the 7 professionals named in the competent team flowcharts was dissatisfied with cooperation, communication and feedback.

### Sensitivity analysis

The t-test revealed no differences on average domain scores between panel members also filling out questionnaire 5 and panel members that filled out questionnaires 1 to 4 only. The analysis of variance with the factors ‘occupation’ (executive, physician, nurse) and ‘setting’ (dispatch, prehospital care and emergency department) revealed no significant findings on ‘mean Likert score’, ‘empty’ and ‘no response’. These results indicate a thorough consensus across settings and occupations, and a minimal bias caused by missing values or selective withdrawal. Table 4 summarises all significant effects of the panelists’ setting on average percentages of ‘no opinion’, distinguished by the domain of the Likert items. Respondents from the emergency department chose the ‘no opinion’ option more frequently on questions about dispatch. Dispatch respondents chose the ‘no opinion’ option more frequently on questions about the prehospital setting and the prehospital/emergency department. These findings indicate that the influence of the ‘non-expert’ answers is small.

**Table 4:** Effects of the work setting on the average percentage of ‘no opinion’ categorized by the domain of the Likert items

		***Average percentage ‘no opinion’ on items in the domain:***		
		**Dispatch**	**Prehospital and emergency department**	**Emergency department**
		**n=33**	**n=67**	**n=236**
**Setting**	Dispatch centre	0.35%	6.31%	11.31%
	Prehospital care	0.97%	0.57%	4.60%
	Emergency department	4.24%	1.35%	1.79%

On dispatch items: p= 0.018 between emergency department and dispatch setting, p=0.013 between emergency department and prehospital setting.

On prehospital and emergency department items: p=0.009 between dispatch and prehospital setting, p=0.022 between dispatch and emergency department.

On emergency department items: p= 0.019 between dispatch and prehospital setting, p= <0.000 between dispatch and emergency department setting.

## Discussion

In this Delphi study, a representative national expert panel achieved consensus on a set of guidelines describing the optimal process of care in the Netherlands for severely injured trauma patient.

According to the guidelines, the time interval between the call to the dispatch nurse until the moment that the patient leaves the trauma room of a level 1 trauma centre should not exceed 60 min. Critical actions in this time frame include the dispatch of an ambulance in conjunction with an MMT, ABCDE stabilisation, formulation of a provisional diagnosis, transportation to a level 1 trauma centre, complete trauma team presence at the ED when the patient arrives, and a complete trauma series of X-rays to confirm or adapt the provisional diagnosis. The Delphi panel indicated that the patient should be cared for by competent teams, as assessed by novel flow charts. Moreover, the perception of cooperation, communication and feedback within and between all team members should be judged as satisfactory.

An inherent limitation of our design is that studies based on expert opinion are low in the ranking of scientific evidence. The relation between the developed process guidelines and functional outcome or mortality should be established in later validation studies. This approach has been applied in the USA, where a national panel of trauma experts reached consensus on 28 criteria to evaluate prehospital trauma care [25]. Subsequently, two of the proposed audit filters were validated, and only one of these filters was associated with increased mortality [26].

A second limitation is that consensus was based on a national study, reflecting the unique aspects of the Dutch setting (including densely populated cities and relatively short distances between hospitals). However, the Dutch trauma system operates according to international standards and many core results might be transferred to trauma systems working with similar processes in a similar structure. Furthermore, some of the guidelines may be generically applicable, as the anatomic and physiological damage resulting from injuries are similar worldwide. However, to assess which of the guidelines may be generalisable to all trauma systems, an international expert panel study is recommended.

A third limitation of our study is that the response rates dropped from 87% in the first round to 48% in the final 5^th^ round; however, the sensitivity analysis did not reveal any selective withdrawal by occupation or by setting. Moreover, the 61 respondents that filled out all 5 questionnaires did not answer questions differently from the 66 respondents that filled out questionnaires 1 to 4 only. This implies that response bias is minimal and that the results of this study can be interpreted as a national consensus among all professions involved in Dutch regional trauma care.

Although national consensus was reached, more research is needed to elucidate the possible connection between time intervals and outcome in the trauma patient. Some authors reported that mortality increased with increasing time spent out of hospital [27,28] whereas others did not find this association [10,29-31]. Authors that studied timeliness in the ED setting could not establish a relation with outcome [10,32]. Our Delphi panel stated that critical limits for the time spent on scene are only relevant for patients in whom a ‘scoop and run’ strategy is applied as this is the subgroup of trauma patients where time is expected to be most crucial; these subgroups include patients with traumatic brain injury and penetrating injuries. Studies on prehospital time intervals in these subgroups could not establish a relation with outcome [22-24].

Although our Delphi study identified many ‘action filters’ that are already addressed in the ACSCOT and ATLS, we also found a filter not yet mentioned in the literature, i.e. formulating a provisional diagnosis in the prehospital setting.

Two of the action guidelines as proposed by our panel are related to the organisation of trauma care: dispatch of an ambulance in conjunction with an MMT and transport to a level 1 trauma centre. Dispatch of an MMT in addition to an ambulance brings a physician to the scene. It is reported that mortality decreases when patients are treated on scene by a physician in conjunction with a nurse [33,34]. Transportation of a severely injured trauma patient to a level 1 trauma centre is in line with the current international standards [9,32,35,36]. In contrast, the establishment of a provisional diagnosis is, as far as we know, never mentioned and/or investigated as a relevant aspect of trauma care. The expert panel agreed on the establishment of a provisional diagnosis as a guideline for quality of trauma care for the prehospital setting and the ED setting. Further research is needed to evaluate the possible value of the provisional diagnosis as a process guideline.

Available evidence on the education and experience of a prehospital or ED team is scarce and inconclusive. Some found a contribution of the surgeon’s experience on outcome of the trauma patient [37,38], whereas others found no effect [39-41]. Available studies were unable to show any effect of trauma courses on the outcome of severe trauma patients [15,16]. No studies were found that combined the competencies of several team members. Future studies should establish whether being treated by competent teams, as assessed with our novel flow charts, offers an advantage in terms of survival and/or functional outcome in the severe trauma patient.

Our panel defined one interdisciplinary process guideline which lies on largely unexplored terrain; its validity in trauma care practice has yet to be established. However, the interdisciplinary process guideline is in accordance with current international standards on medical specialty training that place emphasis on cooperation and communication skills [42].

The results of this study can be seen as a new field standard for quality in trauma care in the Netherlands. The standards are based on a national consensus among all professionals involved in regional trauma care; this should facilitate the acceptance of the guidelines by all stakeholders. Additional steps are required before the set of guidelines can be validated and implemented. First, a thorough analysis of the availability and reliability of the data needed to assess the guidelines is required in order to monitor and improve the performance of a trauma system. Future research needs to determine whether adherence to specific guidelines (or the set as a whole) is associated with improved survival and/or functional outcome of the severe trauma patient.

## Conclusions

The Delphi panel developed 21 process guidelines for trauma care which can be considered as new tools to measure the quality of trauma care in the Netherlands. The time and performance-related process guidelines reflect accepted concepts and are partly underpinned by scientific evidence, whereas the competencies and interdisciplinary-related process guidelines are new in this domain.

In addition to time intervals and appropriate actions, these guidelines emphasise the importance of team competence and the interdisciplinary process in trauma care. The guidelines can be seen as the description of a best practice and a new field standard in the Netherlands. The next step is to implement the guidelines and subsequently monitor the performance of the Dutch trauma system based on these guidelines.

## Abbreviations

ABCDE: Airway Breathing Circulation Disability Exposure and Environment; ACSCOT: American College of Surgeons Committee on Trauma; ATLS: Advanced Trauma Life Support; ED: Emergency Department; MMT: Mobile Medical Team; NGT: Nominal Group Technique.

## Competing interests

All authors have completed the Unified Competing Interest form and declare that all authors (EMH, EFB, JCG, PDB & JJLMB) have no financial interests that may be relevant to the submitted work.

## Authors’ contributions

JJLMB obtained research funding and supervised the conduct of the study, design of questionnaires and data collection. JJLMB, JCG and EMH designed the study. PDB provided statistical advice. EMH recruited the Delphi panel, designed questionnaires, processed responses and analysed data. EMH drafted the article. EFB assisted in drafting the article. All authors contributed substantially to its revision. EMH takes responsibility for the paper as a whole. All authors read and approved the final manuscript.

## Pre-publication history

The pre-publication history for this paper can be accessed here:

http://www.biomedcentral.com/1472-6963/13/79/prepub
